# Spinal epidural abscess: a rare complication of ulcerative colitis after ileal pouch anal anastomosis

**DOI:** 10.1186/s40792-016-0253-3

**Published:** 2016-11-07

**Authors:** Mikio Kawamura, Toshimitsu Araki, Yoshiki Okita, Satoru Kondo, Takashi Ichikawa, Hiroyuki Fujikawa, Keiichi Uchida, Yasuhiko Mohri, Masato Kusunoki

**Affiliations:** Department of Gastrointestinal and Pediatric Surgery, Division of Reparative Medicine, Institute of Life Sciences, Mie University Graduate School of Medicine, 2-174 Edobashi, Tsu, Mie 514-8507 Japan

**Keywords:** Ulcerative colitis, Spinal epidural abscess, Ileal pouch anal anastomosis

## Abstract

**Background:**

Spinal epidural abscess is a rare condition with high morbidity and mortality, for which a delay in diagnosis and treatment can lead to irreversible neurologic deficit or even death. Although patients with spinal epidural abscess have systemic predisposing immunocompromised conditions, spinal intervention, or trauma, this condition has been reported as a result of perforation or fistulization arising from inflammatory bowel disease. We describe herein a rare case of spinal epidural abscess as a complication of ileal pouch anal anastomosis.

**Case presentation:**

A 37-year-old man who had previously undergone restorative proctocolectomy and ileal pouch anal anastomosis for ulcerative colitis presented with complaints of persistent low-grade fever and lumbago with unusual sensation in the lower legs. After evaluation by Gastrografin contrast radiography, computed tomography, and magnetic resonance imaging, he was diagnosed with a spinal epidural abscess extending from L5 to S1. In addition, the abscess communicated with the ileal pouch. He underwent surgical drainage of the abscess, excision of the fistula, and defunctioning ileostomy. Although a second operation for drainage was required for residual presacral abscess, there was no sign of recurrence of the spinal epidural abscess. He eventually was able to close his stoma.

**Conclusions:**

Although spinal epidural abscess is a rare complication, one should take this condition into account when patients complain of back pain or neurologic symptoms of the lower extremities, given the possibility of fistulous communication between the ileal pouch and spine.

## Background

Fistulization is a rare complication of ulcerative colitis during its natural course. However, approximately 5 % of patients after ileal pouch anal anastomosis (IPAA) experience fistula or abscess formation related to the ileal pouch [1–3]. Pouch-related fistula, once formed, tends to be refractory to various treatments. In severe cases, excision of the ileal pouch and permanent ileostomy are unavoidable. Spinal epidural abscess is a rare condition that sometimes causes neurologic disorders. Here, we report a case of spinal epidural fistula extending from the ileal pouch to the spinal epidural space, which we successfully treated without excising the ileal pouch.

## Case presentation

A 37-year-old man complained of lumbago that had persisted for 4 months. He had been diagnosed with ulcerative colitis at the age of 29 years, and had undergone restorative proctocolectomy and hand-sewn IPAA when 33 years old for ulcerative colitis refractory to medical treatment and suspicion of dysplasia. Pathologic evaluation revealed no malignancy in the resected specimens. After IPAA, he had a history of chronic pouchitis, which required continuous antibiotic therapy.

**Fig. 1:**
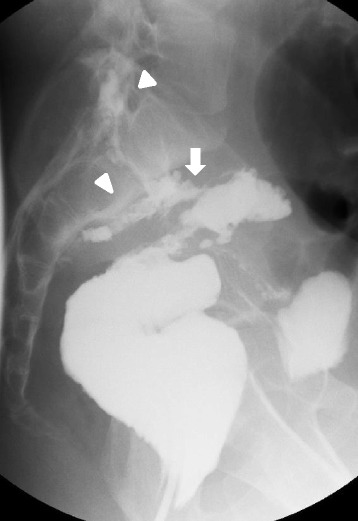
Gastrografin radiographic examination shows leakage from the top of the ileal pouch (*white arrow*) to the spinal canal at the L5–S1 level, forming a spinal epidural abscess (*white arrowheads*)

**Fig. 2:**
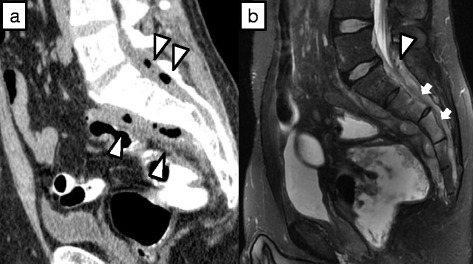
**a** Sagittal CT shows air density in the spinal canal and soft-tissue density anterior to S1 and S2 (*white arrowheads*). **b** T2-weighted MR image shows fluid and gas in the epidural space (*arrowhead*), and enhancement of S3 and S4 (*arrows*), indicating inflammatory extension

Although he had persistent low-grade fever and bilateral paresthesia of the outer thigh, the patient did not complain of weakened muscles apparent in the lower legs. He had no complaints of urinary retention or fecal incontinence. Laboratory tests disclosed elevated inflammatory status (C-reactive protein 3.68 mg/L, white blood cell count 11,780/μL). Gastrografin contrast radiography of the ileal pouch revealed contrast leakage from the ileal pouch extending into the L5/S1 epidural space (Fig. 1). The fistula was isolated from the anastomotic site, located at the top of the efferent limb. A computed tomography (CT) scan of the abdomen and pelvis revealed collection of epidural fluid at L5/S1 (Fig. 2a). Magnetic resonance imaging (MRI) confirmed the CT findings, and the fluid collection at the sacrum did not compress the spinal cord (Fig. 2b). He was diagnosed with spinal epidural abscess attributable to a fistula arising from the ileal pouch.

The patient was admitted to our department for drainage of the spinal epidural abscess. In this case, where causative bacteria should be of enteric origin, we used cefmetazole for empirical antibiotic treatment to cover gram negative bacilli and anaerobes. At surgery, the fistula was observed to originate at the staple line of the top of the pouch in efferent limb and communicated with the promontory of the pelvis. After excising the root of the fistula, suspected to be a possible source of spinal infection, the foramen was closed and a diverting loop-ileostomy procedure was performed.

**Fig. 3:**
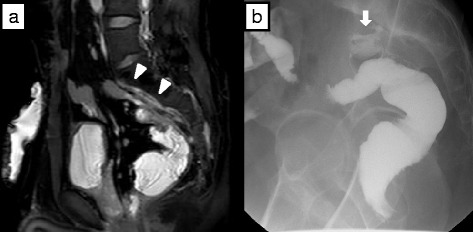
**a** T2-weighted MR image reveals soft-tissue intensity with enhancement anterior to S1–S3 (*white arrowheads*). **b** Lateral radiograph during Gastrografin enema shows extravasation of contrast material from the top of the pouch to the S1–S2 presacral space (*white arrow*), indicating the existence of residual abscess. There is no communication between the abscess and epidural space

Although abdominal distension caused by postoperative ileus persisted, his postoperative course was generally favorable, and he was discharged after 28 days. Ten months after surgery, laboratory tests showed an elevated white blood cell count (11,270/μL) and C-reactive protein level (3.61 mg/L), leading us to suspect a residual abscess. MRI and Gastrografin radiographic examination showed a fistula arising from the ileal pouch to the presacral space, but a spinal epidural abscess could not be detected (Fig. 3a, b). The patient was diagnosed with residual presacral abscess and fistula formation, and underwent further drainage of the presacral abscess. At surgery, there was dense fibrosis and tissue granulation between the top of the pouch and the sacrum (promontory-S2). From the abscess, a fistula extended to the top of the ileal pouch, which was confirmed using pouchoscopy. After mobilizing the ileal pouch from the presacral abscess, the fistula of the pouch was excised thoroughly. As the ileal wall appeared intact, primary closure and defunctioning ileostomy were performed. Postoperatively, the fistula and abscess healed completely. The patient finally underwent closure of the ileostomy 15 months after the initial surgery. Although he requires medication to control pouchitis, he is doing well as an outpatient with no signs of recurrence 3 years post-initial surgery.

## Conclusions

Spinal epidural abscess is an uncommon disease that accounts for 0.2–1.2 cases per 10,000 hospital admissions, with a relatively high rate of associated morbidity and mortality [2, 4, 5]. Most patients with spinal epidural abscess have systemic predisposing conditions, such as diabetes mellitus, renal disorder, human immunodeficiency virus infection, malignancy, morbid obesity, long-term corticosteroid use, alcoholism, or a distant site of infection [2, 6]. Some local conditions are also known as predisposing factors, including spine trauma, spinal surgery, and extrathecal injection or catheter placement into the vertebral canal [2, 6]. In terms of neurologic symptoms and its progression, spinal epidural abscess is divided into four stages: stage 1, back pain, fever, and local tenderness at the level of the affected spine; stage 2, signs of spinal irritation and neck stiffness; stage 3, motor weakness, sensory deficit, and bladder and bowel dysfunction; and stage 4, complete paralysis [7].

Because epidural infection can injure the spinal cord either directly by mechanical compression or indirectly as a result of vascular occlusion caused by septic thrombophlebitis, the associated neurologic dysfunction and mortality are high [6]. Especially in severe cases with neurologic impairment, such as stage 3 or 4 disease, decompressive laminectomy and debridement of infected tissues should be performed immediately [2, 4–6]. Although laminectomy is recommend for the severe case with neurologic impairment, it is impractical to perform decompressive laminectomy along with all spinal epidural abscess because of its invasiveness. Therefore, less extensive surgery might be considered for stage 1 or 2 patients with neurologically mild symptoms [6]. In this case, intestinal communication to spine was apparent and neurologic symptom was relatively mild and slowly progressive. Considering the invasiveness of laminectomy, sufficient drainage of abscess with defunctioning ileostomy and antibiotic therapy might be preferred for this case.

Patients with unexplained persistent or recurrent epidural infection should be carefully inspected for rare sources of infection. In the case of thoracolumbar abscesses, an intestinal–spinal fistula may be causative [6].

Inflammatory bowel disease, especially Crohn’s disease, is complicated by perforation, abscess, and fistula in approximately 30–50 % of cases [8, 9]. Fistula associated with Crohn’s disease sometimes develops between the intestine and other organs [10–12]. In some cases, a fistula from the intestine extends to the epidural space to form a spinal epidural abscess [13–17].

In ulcerative colitis, fistula formation is relatively rare. However, 5–10 % of cases after IPAA have been reported to develop pouch-related fistula [1, 3, 18]. Only one case of ulcerative colitis with IPAA-associated spinal epidural abscess has been previously reported. In this patient reported by Brown et al. [14], although spinal epidural abscess was precisely diagnosed and immediate laminectomy was performed, the infectious source was not detected. Only 3 years later was a fistula extending from the top of the ileal pouch to the presacral space detected. The patient underwent a combined abdominal/perineal pouch excision, and reconstruction of a new pouch with hand-sewn ileal pouch anal anastomosis and construction of a diverting ileostomy.

In the present case, we precisely diagnosed a spinal epidural abscess resulting from an ileal pouch spinal fistula, and assessed the neurologic impairment as stage 2 disease. We performed diverting ileostomy, with thorough drainage and debridement of the abscess. At the initial operation, we mainly intended to eradicate the epidural abscess by fecal diversion. At the second operation, although a presacral residual abscess had been observed, the spinal epidural abscess was cured completely. In line with a previous report that ileal pouch-related pelvic sepsis might be refractory to various treatments [19], we also experienced recurrence of the pelvic abscess. Although reoperation for drainage was required, the patient was finally able to achieve stoma closure.

To the best of our knowledge, this is the first case in the English literature of spinal epidural abscess attributable to an enteroepidural fistula arising from the ileal pouch while retaining intestinal continuity. Although pouch-related spinal epidural abscess is an extremely rare complication of ulcerative colitis after IPAA, one should include this condition in the differential diagnosis when encountering patients with systemic inflammation and neurologic impairment.

## Abbreviations

CT: Computed tomography
IPAA: Ileal pouch anal anastomosis
MRI: Magnetic resonance imaging
